# Multiplexed kit based on Luminex technology and achievements in synthetic biology discriminates Zika, chikungunya, and dengue viruses in mosquitoes

**DOI:** 10.1186/s12879-019-3998-z

**Published:** 2019-05-14

**Authors:** Lyudmyla G. Glushakova, Barry W. Alto, Myong-Sang Kim, Daniel Hutter, Andrea Bradley, Kevin M. Bradley, Nathan D. Burkett-Cadena, Steven A. Benner

**Affiliations:** 1 Firebird Biomolecular Sciences LLC, 13709 Progress Blvd, Box 17, Alachua, FL 32615 USA; 2 Florida Medical Entomology Laboratory, University of Florida, 200 9th Street SE, Vero Beach, FL 32962 USA

**Keywords:** Zika, Chikungunya, Dengue viruses, Reverse-transcription PCR, Luminex DHA, Cationic paper

## Abstract

**Background:**

The global expansion of dengue (DENV), chikungunya (CHIKV), and Zika viruses (ZIKV) is having a serious impact on public health. Because these arboviruses are transmitted by the same mosquito species and co-circulate in the same area, a sensitive diagnostic assay that detects them together, with discrimination, is needed.

**Methods:**

We present here a diagnostics panel based on reverse transcription-PCR amplification of viral RNA and an xMap Luminex architecture involving direct hybridization of PCRamplicons and virus-specific probes. Two DNA innovations (“artificially expanded genetic information systems”, AEGIS, and “self-avoiding molecular recognition systems”, SAMRS) increase the hybridization sensitivity on Luminex microspheres and PCR specificity of the multiplex assay compared to the standard approach (standard nucleotides).

**Results:**

The diagnostics panel detects, if they are present, these viruses with a resolution of 20 genome equivalents (DENV1), or 10 (DENV3–4, CHIKV) and 80 (DENV2, ZIKV) genome equivalents per assay. It identifies ZIKV, CHIKV and DENV RNAs in a single infected mosquito, in mosquito pools comprised of 5 to 50 individuals, and mosquito saliva (ZIKV, CHIKV, and DENV2). Infected mosquitoes and saliva were also collected on a cationic surface (Q-paper), which binds mosquito and viral nucleic acids electrostatically. All samples from infected mosquitoes displayed only target-specific signals; signals from non-infected samples were at background levels.

**Conclusions:**

Our results provide an efficient and multiplex tool that may be used for surveillance of emerging mosquito-borne pathogens which aids targeted mosquito control in areas at high risk for transmission.

**Electronic supplementary material:**

The online version of this article (10.1186/s12879-019-3998-z) contains supplementary material, which is available to authorized users.

## Background

Dengue (DENV), chikungunya (CHIKV), and Zika (ZIKV) viruses are mosquito-borne pathogens that have caused numerous outbreaks in Southeast Asia and more recently undergone geographic expansion in the Americas, causing emerging and serious health problems in humans [1–5]. Although dengue is not new to the Americas, the emergence of CHIKV and ZIKV add to the burden of disease in this region of the world. Endemic to Africa, these arboviruses have rapidly expanded their original geographical range and reached North America and Europe through their exploitation of invasive mosquito vectors [6–8].

The sylvatic transmission cycle of DENV, CHIKV, and ZIKV involves non-human primates and arboreal *Aedes* mosquitoes [9–12]. Domestic container mosquitoes *Ae. aegypti* and *Ae. albopictus* are primarily responsible for transmission of these arboviruses to humans [13–15]. Sufficiently high viremia levels of these pathogens predisposes epidemics in human populations that are sustained by human-mosquito transmission [4, 16, 17] with ZIKV being the first known arbovirus that could be also transmitted directly from human-to-human [18–20]. This latter route of transmission may allow for persistence of ZIKV during times of the year that are not favorable for mosquito proliferation.

The emergence of these medically important arboviruses is associated with geographic expansion of their main mosquito vectors, anthropophilic *Ae. aegypti* [21] and opportunistic, invasive *Ae. albopictus* [16, 22, 23]. Also, high mutation rates among RNA viruses produce the conditions for adaptive evolution to new mosquito species and often gains a high degree of receptivity and infectivity which may facilitate disease emergence. As an example, prior to the emergence of the Indian Ocean strain of CHIKV, *Ae. aegypti* was regarded as the primary epidemic vector with *Ae. albopictus* being secondary in importance. The Indian Ocean strain of CHIKV acquired a single mutation in the envelope protein gene E1 (A226V) that greatly enhanced the vector competence of *Ae. albopictus* [24]. This illustrates that CHIKV adapts locally to vectors, which allows for the possibility of establishing enzootic transmission cycles in new regions. Lastly, human movement and has allowed for enhanced contact rates between infected and uninfected hosts and mosquito vectors. It is this latter mechanism which may have allowed for the emergence of the Asian lineage of CHIKV in the Americas.

The above provides a clear explanation why DENV, CHIKV, and ZIKV co-circulate in the same geographic area infested with competent mosquito vectors, *Ae. aegypti* and *Ae. albopictus*. In this context, cases of patients’ co-infection at least by two viruses (ZIKV/CHIKV or DENV/CHIKV) are reported [25–27], where co-infection occurs via single or multiple mosquito bites [28, 29].

In the absence of specific antiviral drugs and vaccines and in context of given viruses co-circulation and similarity, sensitive diagnostic tools for their detection and discrimination are in great demand.

RT-PCR is regarded as the gold standard for pathogen detection because of its specificity and lower rates of false negatives compared to alternative approaches for arbovirus surveillance such as RAMP (Rapid Analytical Measurement Platform) and the Genie II (OptiGene Co.) [30, 31].

**Fig. 1:**
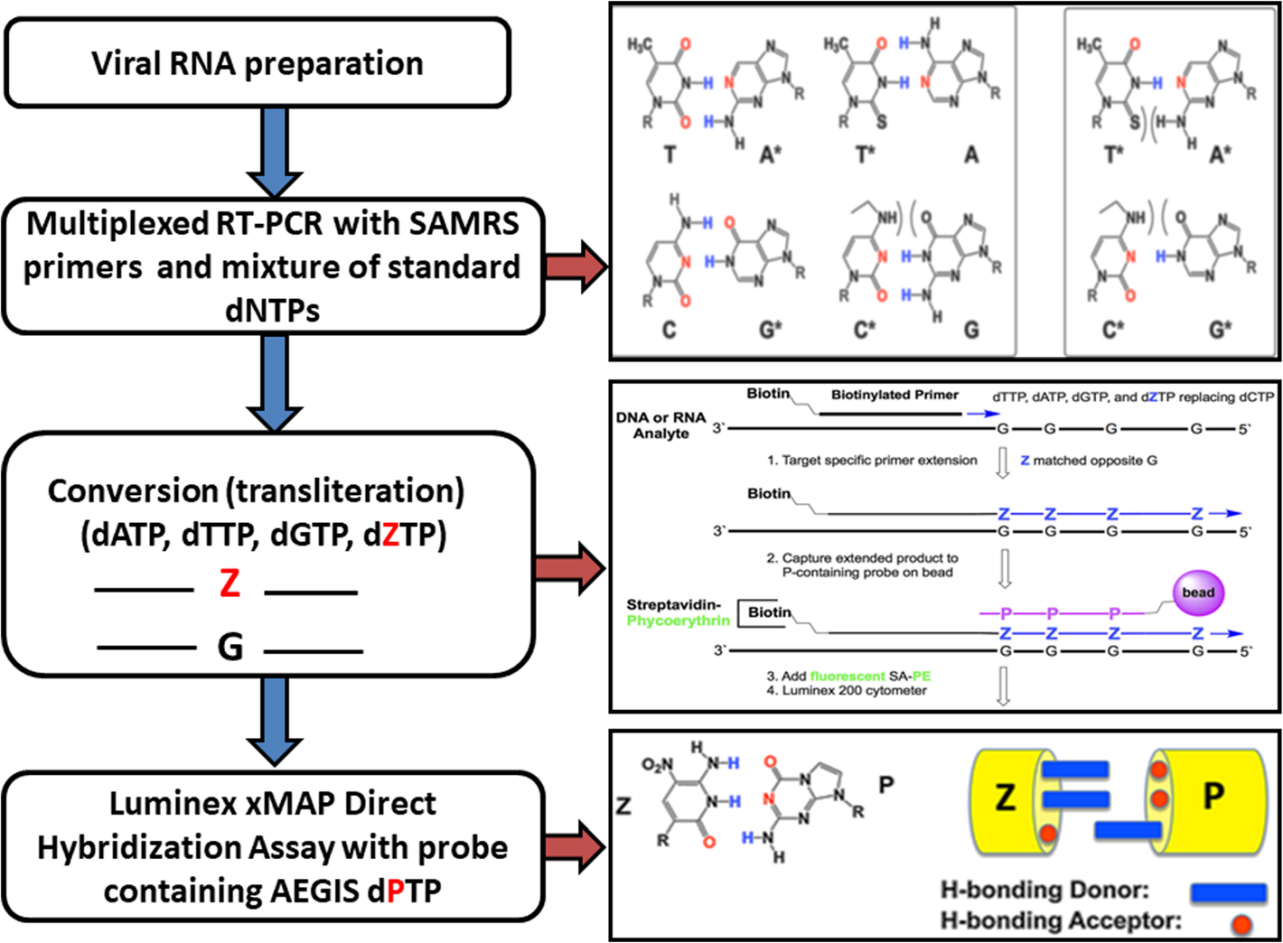
Adapted from [32, 34]. *Left panels****:*** Overview of the Luminex xMAP DHAs protocol developed to detect DENV, CHIKV, and ZIKV RNAs. *Right top panel*: The strategic removal of hydrogen bonding groups gives a self-avoiding molecular recognition system, SAMRS. *Right middle panel*: The “transliteration” strategy allows a template G to direct the incorporation of the AEGIS base Z by primer extension, which exploits the mismatch between G and deprotonated Z. *Right bottom panel*: The artificially expanded genetic information system (AEGIS) adds up to eight nucleotides and contributes four additional base pairs to the four standard nucleotides by strategically rearranging hydrogen bonding patterns. The AEGIS Z: P pair is exploited in this work

Here we present a PCR amplification-based multiplexed Luminex Direct Hybridization Assays (DHAs) diagnostics panel that discriminates DENV1–4, CHIKV, and ZIKV with a low limit of detection of 20 (DENV1) or 80 (ZIKV, DENV2), or 10 (DENV3–4, CHIKV) genome equivalents per assay. Although molecular assays based on PCR-amplification and target-specific hybridization are considered more accurate and sensitive than serological or biological tests, multiplexing often decrease assay resolution [32] and primers dimers usually generate false positives. These obstacles are resolved by a series of innovations from the field of synthetic biology (SAMRS and AEGIS nucleotides), reported previously [32, 33] and shown in Fig. 1 (adapted from [32]) that allowed the elimination of PCR “noise” and increased hybridization efficiency on Luminex microspheres. The assays panel was validated first with all six types of viral RNA (purified from infected Vero cells) and after DENV, ZIKV and CHIKV were detected in a single infected mosquito, in mosquito pools or mosquito saliva (CHIKV and DENV2). DENV2–4 RNA detection was evaluated on the background of nucleic acids from pooled mosquitoes. Infected mosquitoes and saliva were also positively assessed on the surface of a cationic (Q)-paper [34, 35] that bound mosquito and viral nucleic acids via electrostatic interactions.

The multiplexed diagnostics panel presented here combined with a Q-paper capture surface has the potential for a much wider range of applications than only mosquito surveillance, such as the evaluation of clinical samples (serum, urine, whole blood, and amniotic fluid).

## Methods

### Viral strains

#### Oligonucleotides used in this study

Primers and probes (Table 2 and Additional file 1: Table S1) were designed using the “in house” StrainTargeter software package. This procedure was described in our previous publication [32]. PCR-primers built from SAMRS and Luminex probes containing AEGIS nucleotides (Table 2) were synthesized on ABI 394 and ABI 3900 using AEGIS and SAMRS phosphoramides (www.firebirdbio.com). Standard oligonucleotides were purchased from Integrated DNA Technology (IDT, Coralville, USA).

### Mosquito collections and rearing

Laboratory colonies of *Ae. aegypti* from collections in Florida were used in the ZIKV, CHIKV, and DENV1–4 infection studies. Larvae were reared at an approximate density of 150 larvae/L water in plastic trays (25 cm × 30 cm × 5 cm) with water (900 mL) and larval food (0.4 g) consisting of equal amounts of brewer’s yeast and liver powder (by weight) at hatching, and supplemented again with the same amount of food 3–4 days later. Mosquitoes were maintained as described in [34] and allowed to feed on chickens at the Florida Medical Entomology Laboratory once per week to propagate eggs (IACUC protocol no. 201507682). *Ae. aegypti* females laid eggs on damp paper towels in water filled cups. The progeny of these mosquitoes were used for arbovirus infection studies in the biosafety level-3 virology facility at the Florida Medical Entomology Laboratory in Vero Beach, FL.

### Propagation of arboviruses

Isolates of emergent strains of arboviruses (Table 1) were provided by the Centers for Disease Control and Prevention (ZIKV, CHIKV, DENV1–4, MVEV, CEV, LACV, JEV, YFV, MAYV), Florida Department of Health (DENV1), and the University of Texas Medical Branch in Galveston, TX (CHIKV).

**Table 1:** Arboviruses in this study and their viral titer

Family/Genus	Viruses /strains/GenBank	Viral stocks titers, genome equivalents/mL
Flaviviridae/ Flavivirus Group IV positive ssRNA	DENV1 from Key West (2010), BOL-KW010; **GB**: JQ675358	1.9 × 10^8^
	DENV2, New Guinea C (1944), M29898WSV	2 × 10^6^
	DNV3, H-87, (1956), TC00881 WSV	1.6 × 10^7^
	DENV4, H-87, (1956), TC00881 WSV	4 × 10^8^
	ZIKV, Puerto Rico (2015), strain H/PF/2013; **GB**: KU501215.1	1.12 × 10^10^
	JEV, Nakayama-GIII (1935) **GB:** EF531853.1	1 × 10^10^
	YFV, Ghana/Asibi/1927 **GB**: AY640589	4.57 × 10^9^
	MVEV, MVE-1-51, **GB:** AF161266	2.69 × 10^7^
Togaviridae/Alphavirus Group IV, positive ssRNA	CHIKV, La Reunion (2006), Indian Ocean strain (IOC), LR2006-OPY1, **GB:** KT449801	1.89 × 10^8^
	CHIKV, British Virgin Islands (2013), BVI, Asian lineage; **GB:** KJ451624	2.4 × 10^10^
	MAYV, strain TRVL 4675 (1954) **GB**: MK070492.1	3.8 × 10^7^
Peribunyaviridae/ Orthobunyavirus Group V negative ssRNA	CEV, BFS283, Small segment (S), **GB**: U12797) Medium segment (M), **GB**: AF123483	2.69 × 10^10^
	LACV, Original Wisconsin virus (LACV/ human/1960) **GB**: EF485030–EF485032	4.37 × 10^9^

All arboviruses were obtained from human clinical samples of patients residing in or having traveled to geographic regions during outbreaks (Table 1). We created virus stocks by propagating isolates in cultured African green monkey (Vero) cells as described in [35]. Viral titer was determined by plaque assay using procedures similar to established techniques [36–38]. Propagation of ZIKV, CHIKV, and DENV1–4 for infectious blood meals was accomplished by inoculating confluent monolayers of Vero cells in tissue culture flasks (175 cm^2^) with diluted stock. Following a 1-h incubation period at 37 °C with a 5% carbon dioxide atmosphere, media (25 mL) was added to each T-175 cm^2^ flask with cells. Media from cell cultures were harvested following an incubation period (CHIKV, 2-days; ZIKV, DENV1–4, 7-days).

### Dengue, chikungunya, and Zika infected mosquitoes

Cohorts of 50 adult female mosquitoes aged 10–12 days old were placed in cylindrical cages (height x diameter: 10 cm by 10 cm) with mesh lids and allowed to feed for 1 hour on arbovirus-infected blood from an artificial feeding system (Hemotek, Lancashire, United Kingdom) with hog casing membranes. Mosquitoes were deprived of sucrose 24 h before feeding trials. Infectious blood meals consisted of defibrinated bovine blood (Hemostat Laboratories, Dixon, CA) and media from infected cell cultures. Adenosine triphosphate (0.005 M) was added to the infected blood as a phagostimulant [39]. Following feeding trials, mosquitoes were anesthetized at 4 °C and sorted based on observed meal sizes [40]. Fully engorged females were retained and kept at 30 °C and a photoperiod of 14:10 light:dark hours for a length of time that approximated the extrinsic incubation period (ZIKV, 14 days (Zimler and Alto, unpublished data); CHIKV, 6 d [40]; and DENV1–4, 14 days [41]. The extrinsic incubation period is the time from acquisition of the pathogen (ingestion) until the time when transmission (by bite) is possible, measured in days. Adults were provided with 10% sucrose solution from cotton pads. After specified incubation periods, females were tested for transmission potential by the presence of virus in its saliva [41] or individually stored in 2.0 mL centrifuge tubes at − 80 °C until tested for virus infection. Mosquitoes were dissected to separate the bodies from the legs, which were tested separately as indicators of susceptibility to infection and disseminated infection.

### Saliva from chikungunya infected mosquitoes

Seven days after ingestion of DENV-2 and CHIKV-infected blood, females were individually transferred to 37-mL plastic tubes and saliva was collected as described in [35]. The chosen time to collect saliva was based on maximizing the amount of expectorated viruses in saliva [41−43]. The honey was dyed with blue food coloring to provide a visual marker indicating that mosquitoes ingested honey and expectorated saliva onto the Q-paper [41, 44]. Mosquitoes were examined as described in [35] for blue in their crop after 24 h. Mosquitoes and Q-paper were stored at − 80 °C, and Q-paper was tested for the presence of DENV2 or CHIKV RNA for mosquitoes that fed on blue honey.

### Nucleic acid isolation from arboviral stocks and mosquito tissues

Bodies and legs of individual mosquitoes were homogenized separately and viral RNA was isolated as described in [34, 35]. Quantitative RT-PCR for the presence of arboviral RNA was determined as in [35], using the Superscript III One-Step qRT-PCR with Platinum® Taq kit (Invitrogen, Carlsbad, CA) with the CFX96 Real-time PCR Detection System (Bio-Rad Laboratories, Hercules, CA). Primers and probe sets synthesized by IDT (Integrated DNA Technologies, Coralville, IA) had the following sequences:

- ZIKV [35]:
  - Forward Primer, 5′- CTTCTTATCCACAGCCGTCTC-3′
  - Reverse Primer, 5′- CCAGGCTTCAACGTCGTTAT-3′
  - Probe, 5′−/56-FAM/AGAAGGAGACGAGATGCGGTACAGG/3BHQ_1/− 3′
  - The program for qRT-PCR consisted of a 30-min step at 50 °C linked to a 40-cycle PCR (94 °C for 12 s and 58 °C for 60 s).
- CHIKV [34]:
  - Forward Primer, 5′-GTACGGAAGGTAAACTGGTATGG-3′
  - Reverse Primer, 5′-TCCACCTCCCACTCCTTAAT-3′
  - Probe, 5′−/56-FAM/TGCAGAACCCACCGAAAGGAAACT/3BHQ_1/− 3′
  - The RT-PCR assay consisted of a 30-min RT step at 50 °C linked to a 40-cycle PCR (94 °C for 10 s and 60 °C for 60 s).
- DENV:
  - Forward Primer, 5′-GACACCACACCCTTTGGACAA-3′
  - Reverse Primer, 5′-CACCTGGCTGTCACCTCCAT-3′
  - Probe, 5′−/56-FAM/AGAGGGTGTTTAAAGAGAAAGTTGACACGCG/3BHQ_1/− 3′
  - The RT-PCR assay consisted of a 30-min RT step at 60 °C linked to a 40-cycle PCR (95 °C for 15 s and 60 °C for 60 s). Viral stocks were treated similarly, but without homogenization.

Presence of arboviral RNA in the legs of mosquitoes indicate a disseminated infection, a prerequisite for transmission potential [45]. Bodies of mosquitoes with disseminated infection were squished onto the Q-paper and aqueous ammonia (50 μL 1 M) was added on to the carcass, and adsorbed through the Q-paper and allowed to dry at 22–24 °C for 20 min and then frozen at − 80 °C. Mosquito saliva samples were prepared similarly on Q-paper. Mosquito samples and saliva were shipped to Firebird Biomolecular Sciences, LLC for further arbovirus testing as described below.

### Preparation of cationic paper (Q-paper) and measuring its binding capacity

Q-paper was produced by a modification of a procedure from [46]. Its binding capacity was estimated using 1, 3, 5-benzenetricarboxylic acid. Both protocols were presented previously [34, 35].

### Conventional reverse transcription (RT) PCR

RT-PCR was performed with purified viral RNA or infected mosquito total nucleic acids (NA) using SuperScript One-Step RT-PCR with Platinum Taq (Thermo Fisher Scientific, Carlsbad, CA). Each reaction was set up accordingly to the manufacturer’s protocol. Typically, Forward or Reverse primers were added in the reaction mixture to a final concentration of 0.3 μM. To optimize the reaction, additional MgSO_4_ (1.5 mM) was added to the RT-PCR mixture, increasing the final magnesium concentration to 2.7 mM. Cycling conditions from [35] were optimized for a six-fold multiplexed PCR-format: one cycle of the cDNA synthesis and pre-denaturation (53 °C for 30 min and 94 °C for 2 min), 35 cycles of PCR (94 °C for 15 s, 54 °C for 30 s, and 70 °C for 30 s) and final extension at 72 °C for 5 min.

### Transliteration

Reverse primer extension reaction (RPER) [32, 47] was performed with each PCR-amplicon to aid incorporation of 5′-biotinylated reverse primers and convert dCTPs into dZTPs in the resulting amplicon (“transliteration”) as described in [34].

The RT-PCR or RPER were incubated in DNA Engine® Multi-Bay Thermal Cyclers (BioRad, Hercules, CA, USA) or in miniPCR® Thermal Cycler (Carolina Biological Supply Company, Burlington, NC). The latter is a small, portable unit that can be used in the field. Its PCR software downloads to any smart device (as smart phones) running operating systems such as Macintosh® OS, Windows®, or Android™. The program also allows the user to monitor and to analyze the process in real time.

### Luminex direct hybridization assay (DHA)

The dPTP-containing capture probes with an amino-C12 linker at their 5′-ends were coupled to Luminex MicroPlex carboxylated microspheres (Luminex, Austin TX) as described in [32, 34]. Beads were suspended in Tris-EDTA (pH 8.0) to a final volume of 100 μL and counted with a light microscope.

Luminex direct hybridization assays (DHAs) [48] were performed using a “no wash” protocol as described previously [32, 34] with a few modifications. Briefly, aliquots (5 μL) of each RPER (section 2.10) were transferred to 96-well plates (PCR thermo polystyrene; Costar Technologies, Coppell TX). Microspheres were briefly vortexed and sonicated for 20 s, and 2500 of each microsphere types coupled to the target-specific probes were added to the 2 X hybridization buffer (25 μL of 2 X Tm buffer; 0.4 M NaCl; 0.2 M Tris; 0.16 Triton X-100, pH 8.0). The total volume was adjusted to 50 μL with 20 μL sample buffer (10 mM Tris, 05.mM EDTA, pH 8.0). Sample buffer (25 μL) was then added to each background well (negative control). Hybridization was performed at 57 °C followed by the Luminex DHA protocol: 95 °C for 5 min, cool to 57 °C at a speed of 0.1 °C/second, 15 min at the hybridization T 57 °C. Hybridization buffer (25 μL of 1XTm) containing streptavidin-R-phycoerythrin at final concentration of 0.3% (PJRS14, PROzyme, Hayward CA) was added to each hybridization mixture and incubated at 57 °C for 15 min. Each hybridization reaction was triplicated, and “no-target” controls were run in replicates of five. All assays were analyzed for internal bead color and R-phycoerythrin reporter fluorescence using a Luminex 200 analyzer (Luminex xMAP Technology) and the xPonent Software solutions. The median reporter fluorescence intensity (MFI) was computed for each microsphere type in the sample (six totals). The instrument’s gate setting was established before the samples were run and maintained throughout the course of the study.

## Results

### Development of six-fold multiplexed RT-PCR based Luminex DHA platform for detection of four dengue serotypes, chikungunya, and Zika viruses and validation of panel sensitivity

Viral RNA stock solutions were purified from viral isolates propagated in Vero cell culture, tittered, and expressed as genome equivalents.

**Fig. 2:**
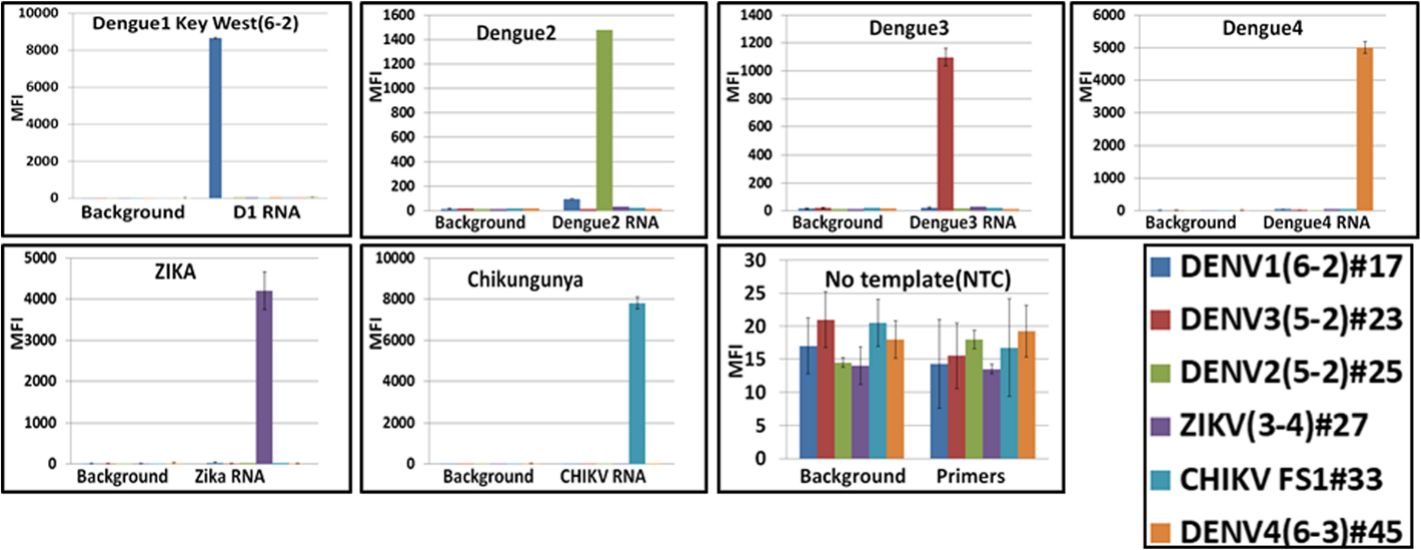
The six-fold multiplexed xMAP Luminex diagnostics panel for detection of DENV1–4, CHIKV, and ZIKV in mosquitoes was developed using viral RNAs (Table 1). Luminex DHA profiles are presented: each DHA was performed with biotinylated viral PCR-amplicon and six target-specific, dPTP-containing probes. The SAMRS technology was employed for RT-PCR, and AEGIS technology for molecular hybridization on Luminex beads. MFI, median fluorescent intensity (Luminex unit); Background, sample buffer was added to the wells; NTC, no template control, ddH_2_0 was added to RT-PCR mixture; *lower right panel* presents Luminex bead identities. Each bead annealed to a target-specific probe: for example, D1 (6–2) #17 stands for DENV1 (D1) probe (6–2) annealed to Luminex bead #17

Several sets of primers and probes were designed to target each viral RNA (Additional file 1: Table S1). First, all standard primers and probes were tested by monoplexed conventional RT-PCR followed by a downstream hybridization on the Luminex platform. Next, six-fold multiplexed assays were assembled and performed with viral RNAs and combinations of primers/probes that improved assay performance (data not shown). Finally, the oligonucleotides that contributed to assay sensitivity (the lowest detection limit) and specificity (didn’t cross react with any other viruses of the assay panel) were selected and their counterparts containing SAMRS and AEGIS nucleotides were synthesized (Table 2) and tested with DENV1–4, CHIKV, and ZIKV RNAs. For this, one-step RT-PCRs (20 μL each) were performed with 1 μL of viral RNA (each RNA input is shown in Table 3). An aliquot of each of the double-stranded amplicons (2.5 μL of each PCR) were transferred into the reverse-primer extension/transliteration reaction (20 μL) to favor the production of single-stranded biotinylated amplicons containing the AEGIS base dZTP. The final products were hybridized, on a liquid Luminex platform, against six types of Luminex beads, each annealed to target-specific probes containing the complementary AEGIS base dPTP (Fig. 1). Beads were analyzed for specific fluorescence (median fluorescence intensity, MFI), generated by the R-phycoerythrin reporter. Fluorescence was produced only by double-stranded amplicon-probe hybrids bound to beads of known identities. All assays (6 repeats for each RNA tested) were positive and specific, generating a strong and clear fluorescence signal (MFI), while a “no template” negative control remained at background level (Fig. 2).

**Table 2:** PCR primers and Luminex probes in this study. All reverse primers are 5′- biotinylated. All probes have 5′-amino-C12-modified. P, AEGIS nucleotide; *, SAMRS nucleotides. R, mixed A, and G bases. Position of primers and probes refer to the chosen sequence of the viral strains

Oligonucleotide identity	Oligonucleotides sequences	Position, nucleotides bases	GB accession number	Targeting gene/ region
DENV1 Forward primer	GGCC**R**GATTAAGCC*A*T*A*G	10,267–10,349	KY926849.1	UTR
DENV1 Probe	A**P**A**P**CTAT**P**CT**P**CCT**P**T			
DENV1 Reverse primer	GCTTTCGGCCTGA*C*T*T*C			
DENV2 Forward primer	CGTGTC**R**ACTGT**R**CA*A*C*A*G	17–136	KY461768.1	Capsid/pre-membrane protein gene
DENV2 Probe	ATTCTCACTT**PP**AAT**P**CT**P**C			
DENV2 Reverse primer	A**R**TATCCCTGCTGTT*G*G*T*G			
DENV3 Forward primer	AACACTCTGGGAAGGAT*C*A*C*C	7405–7507	KT726361.1	Non-structural Protein NS4B
DENV3 Probe	TT**PP**AACACCAC**P**ATA**P**CT			
DENV3 Reverse primer	AGCAAGCCCAGCT*C*C*T*G			
DENV4 Forward primer	GCAGGCAAAAGCCA*C*A*A*G	3289–3430	AH0119363.2	Non-structural protein NS2A
DENV4 Probe	A**P**T**PP**AC**PPP**ATAACA**P**T			
DENV4 Reverse primer	CATGACCTGCCCTA*A*T*T*G			
CHIKV Forward primer	CAGATGGCAACGAA*C*A*G*G	6083–6273	KY575571.1	Non-structural protein nsP4
CHIKV FS-1 Probe	CCTTT**P**CAA**P**CTCCA**P**ATC			
CHIKV Reverse primer	GGGTCCTCTGAGCT*T*C*T*C			
ZIKV Forward primer	AGGGACCTCCGACT*G*A*T*G	9981–10,112	KY415991.1	Non-structural protein NS5
ZIKV Probe	**P**AAA**PPP**A**P**AAT**PP**AT**P**ACC			
ZIKV Reverse primer	CCTCAATCCACACTCT*G*T*T*C

**Table 3:** Viral RNA used in assays (genome equivalents)

Virus	DENV1	DENV2	DENV3	DENV4	ZIKV	CHIKV, La Reunion	CHIKV, BVI
Viral RNA/ 20 μL assay	1.9 × 10^4^	2 × 10^2^	1.6 × 10^3^	4 × 10^3^	1.89 × 10^4^	2.4 × 10^5^	1.89 × 10^4^

**Fig. 3:**
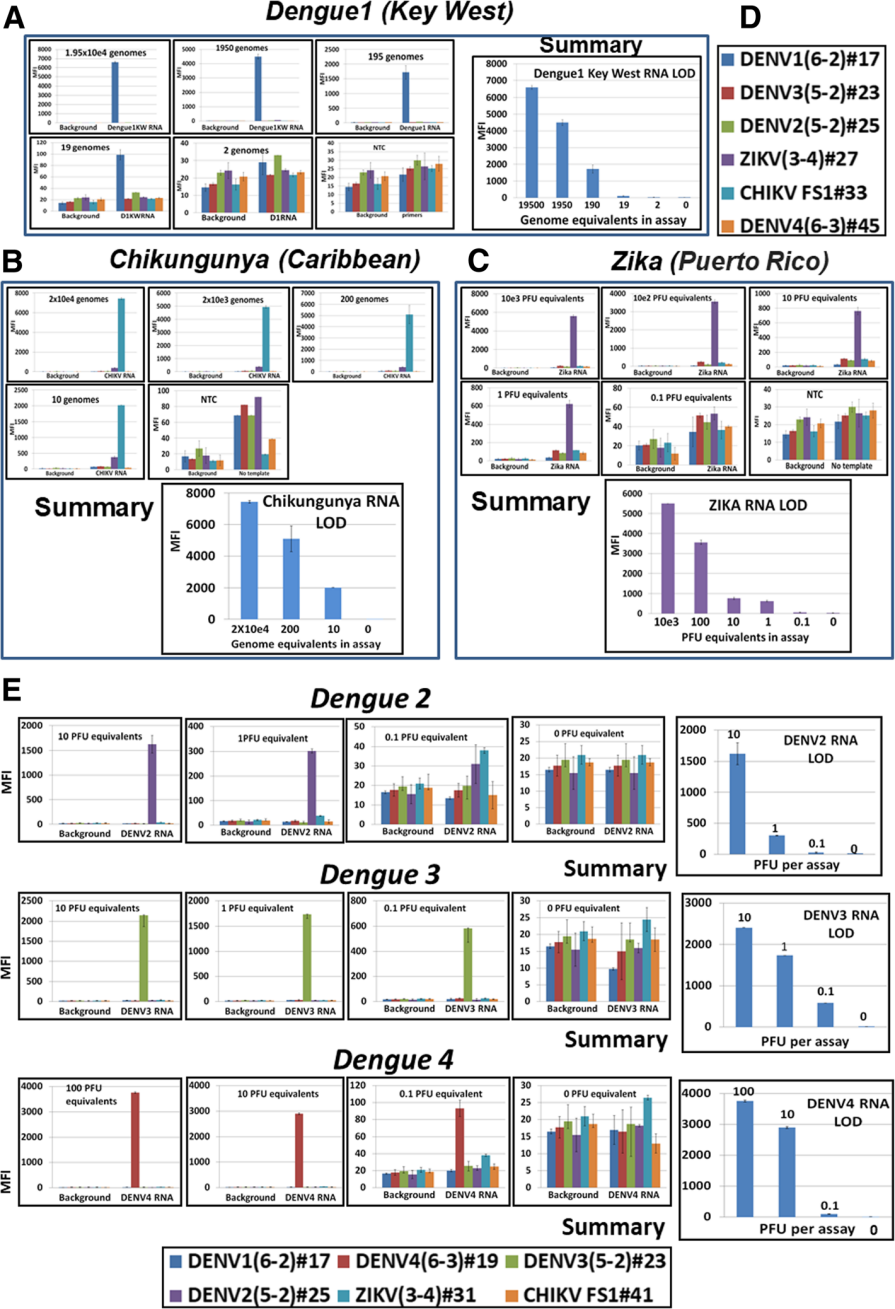
Limits of detection of the diagnostics panel was evaluated with 10-fold serial dilutions of DENV1–4 (**a**, **e**), CHIKV (**b**), ZIKV (**c**) RNAs. Luminex profiles for all dilutions are presented. **d**, Luminex beads/probes identities. MFI, median fluorescent intensity (Luminex unit)

To validate the sensitivity of the diagnostics panel (the level of RNA detection), each viral RNA stock was diluted serially (10-fold steps) and each of the dilutions was evaluated by the panel for the presence of each specific viral RNA (Fig. 3). For this, 1 μL of each RNA dilution was transferred to the six-fold multiplexed RT-PCR, followed by a “transliteration” reaction and, finally, by downstream detection on the six-fold multiplexed Luminex platform. Aliquots from ten independent dilutions were analyzed. The limits of assay detection (LOD) were 10 genome equivalents for DENV3–4, 20 genome equivalents for DENV1, and 80 genome equivalents for ZIKV or DENV2. Next, the diagnostics panel were validated on infected mosquito samples.

### Evaluation of dengue, chikungunya, and Zika viruses in single infected mosquito and mosquito pools with one infected mosquito by a multiplexed diagnostics panel

To confirm the infection, viral titers in leg tissue were evaluated by plaque assays.

Total nucleic acids (NA) were purified from *Ae. aegypti* or *Ae. albopictus* mosquito bodies found to be virus-positive (Table 4) and control mosquitoes (non-infected, *n* = 50). An aliquot (1–3 μL) from each NA preparation (50 μL) was used for specific viral RNA detection. Totally, 50 infected mosquitoes (confirmed by leg titration) were analyzed for each virus tested.

**Table 4:** Examples of viral titers in infected mosquito leg tissue

Virus	Mosquito identity	Leg titer, genome equivalents/mL^a^
DENV1	*Ae. aegypti* #168	1.18 × 10^5^
DENV1	*Ae. aegypti* #169	1 × 10^3^
CHIKV	*Ae. albopictus* #11	1.81 × 10^5^
CHIKV	*Ae. albopictus* #14	0.4 × 10^3^
CHIKV	*Ae. albopictus* #15	1.71 × 10^6^
CHIKV	*Ae. albopictus* #16	6.67 × 10^5^
CHIKV	*Ae. albopictus* #19	8.78 × 10^5^
ZIKV	*Ae. aegypti* #16	4.01 × 10^5^
ZIKV	*Ae. aegypti* #19	3.99 × 10^6^
ZIKV	*Ae. aegypti* #26	1.98 × 10^6^
ZIKV	*Ae. aegypti* #57	7.70 × 10^5^
ZIKV	*Ae. aegypti* #61	6.10 × 10^5^

^a^A lower viral titer is present in mosquito legs than in the whole mosquito body, as described for DENV [49] and CHIKV [60, 61]

**Fig. 4:**
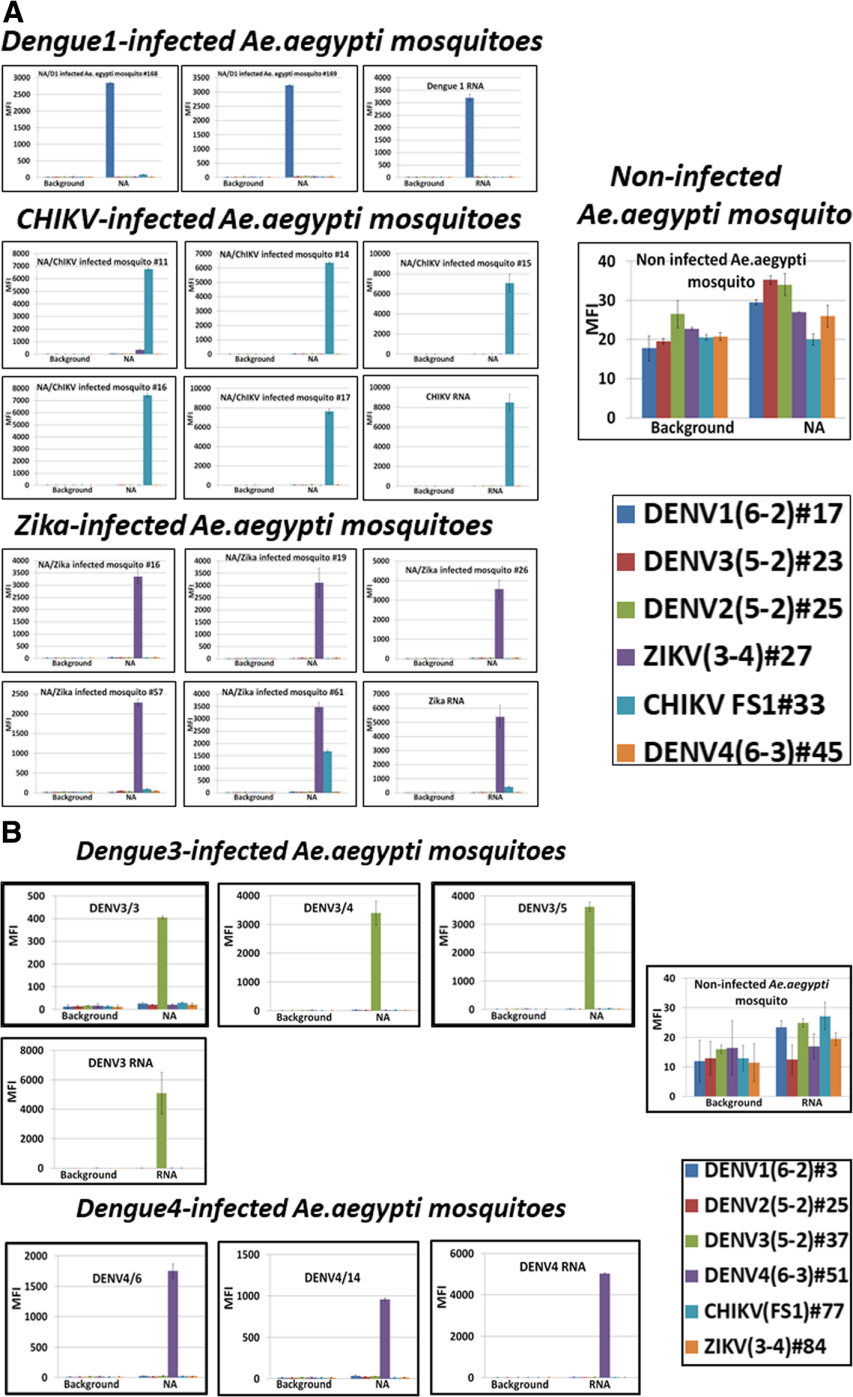
DENV, CHIKV, and ZIKV were detected in single *Ae. aegypti* infected mosquitoes. *Panels* form *left* present Luminex profiles of six-fold multiplexed DHAs for infected mosquitoes and *right top panel* presents representative assay for non-infected mosquito (negative control); *right bottom panel* presents identities of Luminex beads and probes with artificial dPTP

All assays were positive for the appropriate pathogen and generated only strong specific fluorescent signals (in the 3000- to 8000- MFI -units range) (Fig. 4, representative data shown). Two of all samples produced an additional, smaller, non-specific CHIKV signal (as an example, ZIKV-infected *Ae. aegypti* mosquito #61 is presented in Fig. 4). Assays performed with non-infected mosquito NA (negative controls) were at background level and displayed similar Luminex profiles (representative assay in Fig. 4).

**Fig. 5:**
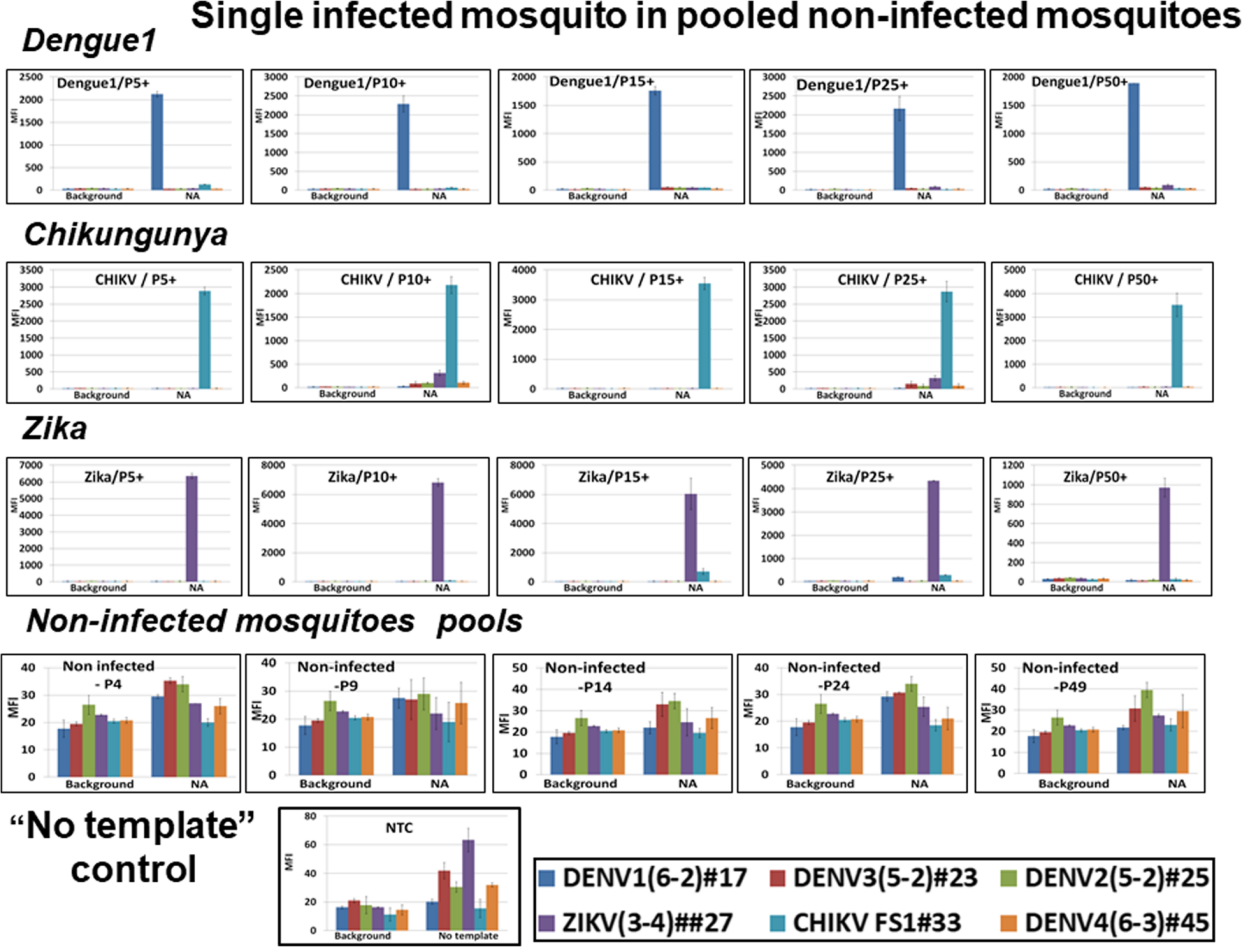
DENV, CHIKV, and ZIKV were detected in mosquito pools (5–50) containing only one infected individual (three rows of *top panels*) but not in pools (4–49) of non–infected mosquitoes (negative controls, *bottom panel*). NTC, “No template” negative control; MFI, median fluorescent intensity

Next, we assembled pools of 5, 10, 15, 25 or 50 *Ae. aegypti* mosquitoes, each with only one DENV1-, ZIKV- or CHIKV-infected individual (P+), and total NA were purified. Infected mosquitoes were confirmed by mosquito “leg” tittering (data not shown). Pools of 9, 14, 24 and 49 non-infected mosquitos (−P) were analyzed in parallel. In total, 10 pools for each combination were analyzed. All assays performed with NA from infected mosquito pools were positive for the given pathogen, and only virus-specific signals were registered on the Luminex platform (for DENV: 2000 to 2500 MFI units, for CHIKV: 2000 to 4000 and for ZIKV: 4000-6000). All assays with NA from non-infected mosquito pools were at background (Fig. 5, the representative data for each group are shown).

**Fig. 6:**
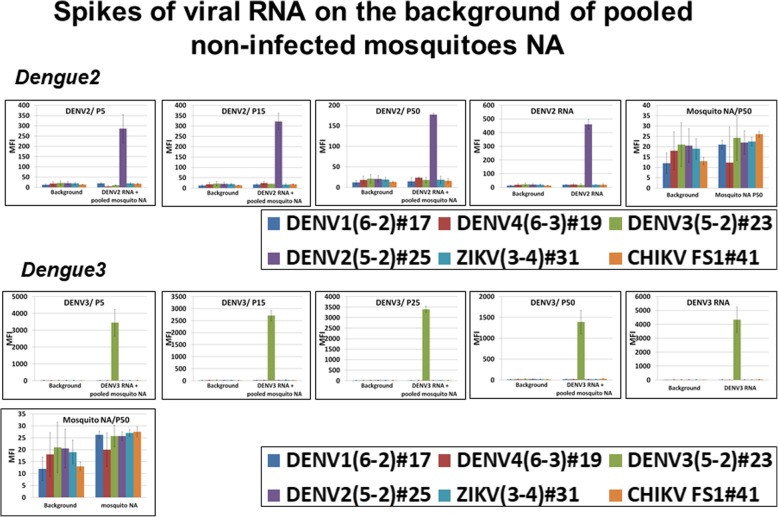
Validation of DENV2–3 RNA spikes (1 PFU equivalent) on the background of pooled mosquitoes’ NA. P5, P15, P25, P50, pools of 5, 15, 25 and 50 *Ae. aegypti* mosquitoes; MFI, median fluorescent intensity (Luminex unit); panels “DENV2 or 3 RNA”, positive assay control, 1PFU of viral RNA was analyzed by multiplexed assay; mosquito NA/50, negative control, mosquito RNA purified from pool of 50 individuals was analyzed by the multiplexed assay

To explore the range of dengue serotypes, DENV2–4 RNA were analyzed within a NA mosquito background by adding (“spiking”) RNA from serotypes 2, 3, and 4. Here, DENV2–4 RNA (100 genome–equivalent, lesser then the content of a single infected mosquito) was mixed with NA purified from pooled non-infected *Ae. aegypti* mosquitoes for assay evaluation. The assay was capable to detect each serotype RNA in given pools (Fig. 6, representative data for each group are shown). DENV-4 RNA was analyzed on the NA background of 15 or 50 *Ae. aegypti* mosquitoes. MFI values obtained in these latest assays were 2000 and 1500 MFI, similar to values registered for DENV-3 RNA. Mosquito background did not interfere with the DENV2–4 RNA; neither false positive signals nor false negative signals were seen.

### Evaluation of viral RNA in infected mosquitoes and saliva on cationic (Q) paper surface by multiplexed diagnostics panel

**Fig. 7:**
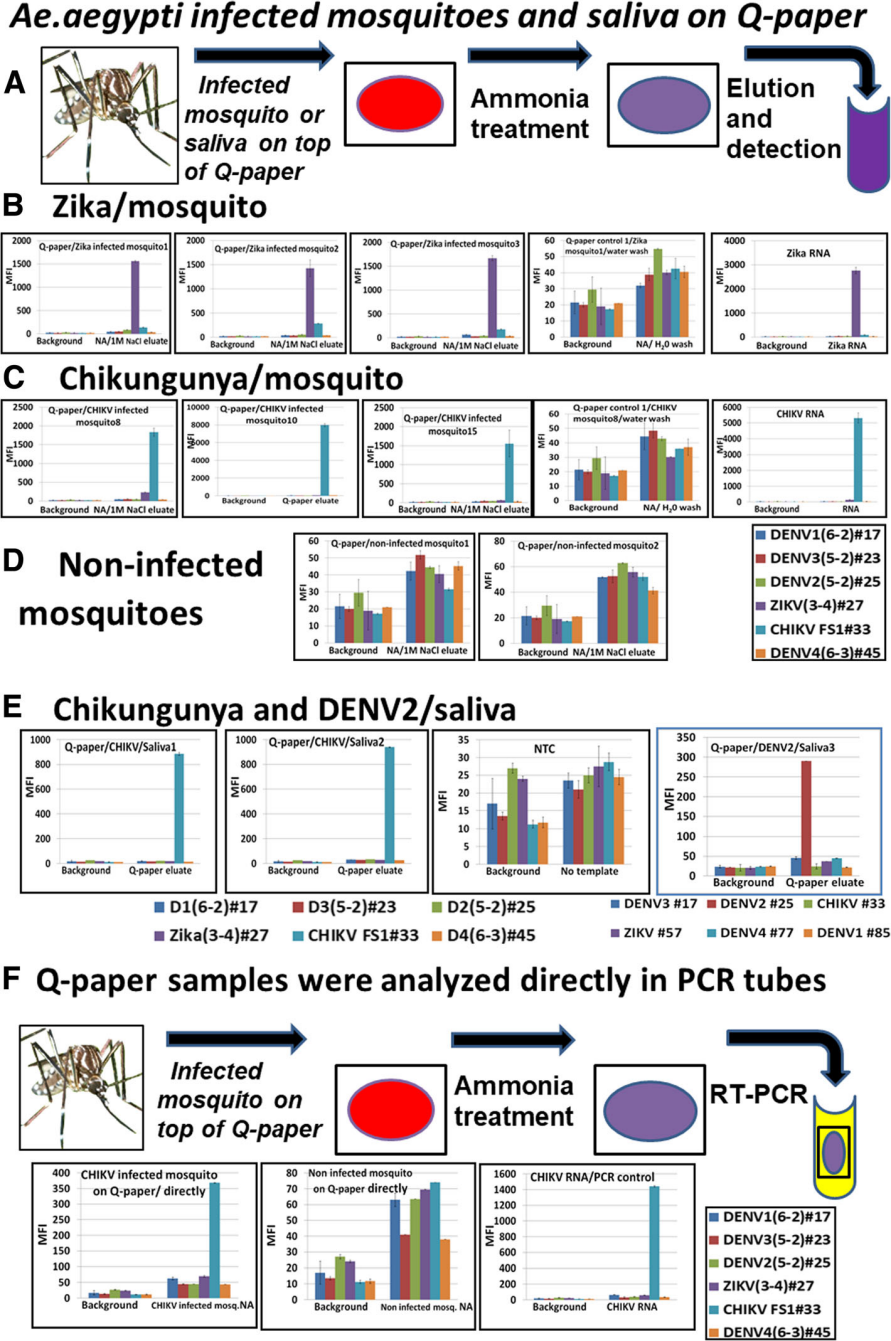
ZIKV- and CHIKV-infected mosquito detection on cationic (Q) paper. **a**, sample preparation and procedure overview; **b**, Luminex profiles of the assays performed with ZIKV-infected mosquitoes and controls; **c**, Luminex profiles of the assays performed with CHIKV-infected mosquitoes and controls; **d**, Non-infected mosquitoes on Q-paper, negative controls; **e**, Saliva from CHIKV and DENV2 infected mosquitoes on Q-paper, two panel on the *bottom* present Luminex beads and probes pairs; **f**, infected mosquito on Q-paper analyzed directly by RT-PCR. Negative control, non-infected mosquito on Q-paper validated directly by RT-PCR. NA, total nucleic acids isolated from infected or non-infected mosquito tissues. *Right bottom panel* presents Luminex beads and probes pairs. Mosquito images by NDBC

Previously [34, 35], we presented the simple technology needed to collect, preserve, and analyze an infected mosquito on Q- paper (overview presented in Fig. 7a). First, *Ae. aegypti* mosquito bodies or saliva infected by CHIKV or ZIKV and non-infected controls were “squashed” on Q-paper and treated with a drop ammonia solution (pH ≥ 12, with added NaOH) to disintegrate the mosquito tissue and release viral RNA. Next, the NAs were eluted with 1 M NaCl solution, and eluates were column-desalted then analyzed by the workflow described above. Each sample infection was confirmed in the same way as mentioned in section above.

With this Q-paper platform, laboratory mosquitoes infected with CHIKV (*n* = 35) and ZIKV (*n* = 25) or saliva from DENV2-infected (*n* = 30) and CHIKV-infected (*n* = 35) mosquitoes were analyzed by (Fig. 7b-c, e (representative data for each group are shown)). All multiplexed assays were positive for the appropriate viral RNA and generated only target-specific and clear fluorescent signals. All negative controls were at background level (Fig. 7d, e). Saliva samples generated lower fluorescence, but agreed with DENV2 or CHIKV loads in saliva samples (“leg” titer about 10^4^ genomes equivalents/mL) (Fig. 7e). Sensitivity of the assays did not differ among the Asian and Indian Ocean lineages of CHIKV.

Some of the Q-paper samples with *Ae. aegypti* mosquitoes infected by CHIKV were not eluted, but analyzed directly on Q-paper (Fig. 6f, *n* = 10, representative data are shown). For this, Q-paper with infected mosquito was cut and the small fragment (2X4 mm) was added directly to the RT-PCR mixture and analyzed by the assays panel. The assay with CHIKV-infected mosquito samples produced target-specific signal (about 400 MFI units). The intensity of fluorescence might correlate with the quantity of RNA on these small Q-paper pieces. Negative control (non-infected *Ae. aegypti* mosquitoes on similar paper fragments (*n* = 8)) slightly exceeded the background level.

### Evaluation of non-related arboviruses from Flaviviridae, Peribunyaviridae and Togaviridae families by multiplexed diagnostics panel

To increase the panel value, additional non-related viruses from *Flaviviridae* (JEV, YFV, MVEV)*, Peribunyaviridae* (CEV, LACV) and *Togaviridae* (MAYV) families (Table 1) were evaluated. Ten, 100, and 1000 genomes of each viral RNA were loaded to the reaction mixtures (*n* = 6 for each viral RNA concentration). Positive controls were DENV1, CHIKV and ZIKV RNA (100 genomes/assay, *n* = 3 for each virus). No template controls were included in each assay group to judge primer dimers.

All non-related viral assays were negative while DENV1, CHIKV and ZIKV generated positive signals (2000 MFI, 4000 MFI and 1000 MFI respectively).

Separately, 100 genome equivalents of each non-related viruses or DENV1, CHIKV, ZIKV (positive controls) were analyzed on mosquitoes background (pool *n* = 15, each assay was triplicates). All assays with non-related viruses were negative. All controls were positive (in a range of 1500–3000 MFI).

## Discussion

Given the uncontrolled invasion of DENV, CHIKV, and ZIKV into non-endemic areas, and in the absence of vaccines and specific antiviral drugs, these mosquito-borne viruses are now having great medical importance worldwide [4, 49].

Because the diseases generated by these viruses are transmitted by competent mosquitoes, sensitive and comprehensive methods are needed for viral surveillance. Such comprehensive diagnostics assays might be also the primary tools to reduce the risk of outbreaks through arbovirus surveillance of their mosquito vectors, enabling targeted mosquito control in high-risk areas.

Due to high specificity and sensitivity, nucleic acid amplification-based tests (NAATs) are valued for pathogen detection and discrimination. Numerous NAATs capable of detecting ZIKV, CHIKV or DENV have been reported [50–53], but few are commercially available (such as BioRad [54], GESIG [55], Altona diagnostic [56] and etc.).

In this study, a comprehensive multiplexed diagnostics panel for detection of these emerging viral pathogens was developed and validated. The panel is capable to discriminate given arboviruses with a limit as low as 10–20 genome equivalents for CHIKV, DENV1, DENV3–4, or up to 80 genome equivalents for DENV2 and ZIKV. The panel backbone is a reverse-transcription PCR amplification followed by the direct hybridization assay on a Luminex platform. To increase sensitivity and performance, artificial nucleotides were used in PCR primers (SAMRS) and Luminex probes (AEGIS). These technologies have been tested and described previously for other targets [32]. In the current study, the introduction of SAMRS primer improved PCR and eliminated non-specific CHIKV signal in DENV1 multiplexed assays with standard primers (data not show).

Next, the diagnostics panel was validated with NA from mosquitoes infected with DENV1–4, CHIKV, and ZIKV or with NA from mosquito pools that included only one infected mosquito. The infected mosquitoes were selected by evaluation of viral titer by qRT-PCR of dissected leg tissue. The multiplexed assays were positive for the appropriate pathogen, and generated strong virus-specific signal when pathogens were pooled with non-infected mosquito samples.

As an additional control group, non-related arboviruses (samples of pure viral RNAs and viral RNAs on mosquito background) from Flaviviridae, Peribunyaviridae and Togaviridae families were evaluated by given panel with only negative outcomes.

We also tested mixed viral RNA, and after combinations of 2–4 mosquitoes infected with either four serotypes of DENV or a single serotype of DENV and ZIKV/CHIKV. All outcomes were positive. Each virus included in assay generated the specific signal but values of the signals varied (data are not shown in this paper). It would be an extremely rare event for single mosquitoes to become naturally infected by three viruses. Therefore, these types of infected mosquito samples are less important overall contributors to arbovirus epidemiology. Moreover, although all given viruses could circulate simultaneously in the same area, only one or two would dominate. It is unlikely that a pool of 25 mosquitoes collected for viral surveillance would contain all six viruses together. It is also obvious that if this happened, the given viruses would be reproduced in mosquitoes species non-equally.

Previously [34, 35], a cellulose-based paper (“Q-paper”), derivatized with quaternary ammonium groups was shown to be a convenient platform to collect (in the field or in the laboratory), preserve, and store mosquitoes or mosquito saliva for downstream detection of pathogens (workflow shown in Fig. 7). Here we coupled the Q-paper technology for mosquito bodies and saliva collection with downstream arbovirus detection by the multiplexed diagnostics panel reported. The Q-paper eluates were positive in all infected samples, with strong specific outcomes on the Luminex platform, while non-infected samples showed background level fluorescence (Fig. 7).

Separately, pathogen from infected mosquito bodies on Q-paper was evaluated directly by PCR (Fig. 7f). These also displayed positive results if an arbovirus was present. The approach described above omitted the need for a NA purification step and simplified the assay procedure.

One limitation of the current study is that we did not compare the performance of our assay against currently available multiplex qPCR assays for arbovirus detection (e.g. Genesig, CDC Trioplex, and AccuPower). It is difficult to judge and compare the efficiency of commercial assays without experiments that make use of aliquots from the same biological samples. In general, we can summarize here, that most of the existing assays are monoplexed (as Genesig assays: CHIKV kit based on real time RT-PCR, capable of detecting about 100 copies of targeted template, and RNA extraction is needed). There are a few commercial assay that are multiplexed, allowing for discrimination between DENV, CHIKV, ZIKV, but not among DENV serotypes. An evaluation of Trioplex real-time RT-PCR (Trioplex assay) in serum, whole blood, and for ZIKV in urine showed a limit of detection of approximately 10^3^ genome copy equivalents/mL [57]. A review of 14 molecular assays for ZIKV, all with emergency use authorization as permitted by the U.S. Food and Drug Administration, showed a range of detection from about 12 to 1.9X10^4^ copies or genomic copy equivalents/mL [58]. Taken together, the current study demonstrates assays that are similar or more sensitive to detection of arboviruses in the multiplex assay. Sensitivity of monoplex and multiplex assays are especially important for ZIKV surveillance given relatively low viral loads in humans (2.7–3.9 log copies/mL in whole blood and 2.2 to 2.8 log copies/mL in plasma [58, 59]. Our design and methodology has the benefit of omission of viral RNA purification. Most of the other assays require RNA purification. In the current study, we coupled a Q-paper tool for mosquito sample collection and storage which allows for omission of the RNA purification step followed by downstream viral RNA detection. We demonstrate that our methods are robust for arbovirus sample collection and detection by RT-PCR. Future work in this area should evaluate how commercially available multiplex qPCR assays for arbovirus detection compare to our assays.

In laboratories equipped with PCR-machine and Luminex instrument, the cost of single assay will not exceeded $15–20 (plus labor cost). The assay flow (Fig. 1) includes three steps: reverse-transcription PCR (about 1.5 h), transliteration (about 20 min) and specific molecular hybridization on Luminex beads (1 h, depending on the amount of sample). So, skilled technicians should be able to successfully complete the assay in about 3–4 h.

In modern version of Luminex instrument all three assay steps can be consecutively executed on Luminex platform (all reagents are loaded in Luminex cartridge and all three reactions performed automatically without spatial separation, minimizing possible contamination). The 96-plate format of Luminex platform is an obvious advantage of our approach that reduces a cost of a single assay if run separately.

In summary, mosquito samples can be field-collected on Q-paper and transferred to a laboratory equipped with Luminex instrument, store and analyzed in a statistically sufficient scope by the multiplexed Luminex-based assay. This technology may be a useful tool for mosquito monitoring and surveillance, especially in areas where dengue, chikungunya, and Zika co-circulate.

This methodological tool based on synthetic biology may be used in other applications as well, such as high-throughput detection of pathogens in clinical samples.

## Additional file

Additional file 1: **Table S1.** PCR primers and Luminex probes designed for this study. R, mixed A and G bases; Y, mixed C and T bases. Oligonucleotides s selected to assemble the diagnostics panel are in *Italic Bold. (DOCX 13 kb)*

## Abbreviations

AEGIS: Artificially expanded genetic information systems
CEV: California encephalitis virus
CHIKV: Chikungunya virus
DENV1–4: Dengue serotypes 1–4 viruses
IACUC: Institutional Animal Care and Use Committee
JEV: Japanese encephalitis virus
LACV: La Crosse virus
MAYV: Mayaro virus
NS: Non-structural
Q-paper: Cellulose-based cationic paper
RT-PCR: Reverse transcription polymerase chain reaction
SAMRS: Self-avoiding molecular recognition systems
xMap Luminex technology: Multi-Analyte Profiling Luminex technology
YFV: Yellow fever virus
ZIKV: Zika virus
