# Supplementary material for: Multiplexed kit based on Luminex technology and achievements in synthetic biology discriminates Zika, chikungunya, and dengue viruses in mosquitoes
Source: BMC Infect Dis. 2019 May 14;19:418. doi: 10.1186/s12879-019-3998-z (PMC6518713; doi:10.1186/s12879-019-3998-z)
Supplement: Supplementary file 1 — Table S1. PCR primers and Luminex probes designed for this study. R, mixed A and G bases; Y, mixed C and T bases. Oligonucleotides s selected to assemble the diagnostics panel are in Italic Bold. (DOCX 13 kb) [file 12879_2019_3998_MOESM1_ESM.docx]

**Additional file 1**

Table S1. PCR primers and Luminex probes designed for this study. R, mixed A and G bases; Y, mixed C and T bases. Oligonucleotides s selected to assemble the diagnostics panel are in ***Italic Bold.***

| Primers and probed identity | Sequences |
| --- | --- |
| DENV1v5-1_Fp1 | TCACACCAYTTCCACCAG |
| DENV1v5-1_DEN1_Probe1 | GGAAATAGTGGTGCCATGC |
| DENV1v5-1_Rp1 | GCCCTRCCCACAAGTTCATC |
| DENV1v5-2_Fp1 | GTGCTTYGAYGGGGAAAG |
| DENV1v5-2_DEN1_Probe1 | GAGAACATGGACGTGGAGAT |
| DENV1v5-2_Rp1 | TCACCTGAGACRCTTCTTC |
| DENV1v5-3_Fp1 | ARAGCTATGCTGCCTGTG |
| DENV1v5-3_DEN1_Probe1 | AGTCAGGCCGAAAGCCA |
| DENV1v5-3_Rp1 | CCACGATGGAGCYACAGG |
| ***DENV1v6-2_Fp*** | ***GGCCRGATTAAGCCATAG*** |
| ***DENV 1v6-2_Probe*** | ***AGAGCTATGCTGCCTGT*** |
| ***DENV 1v6-2_Rp*** | ***GCTTTCGGCCTGACTTC*** |
| DENV1v6-3_Fp | GCGAGAAACCGCGTGTC |
| DENV1v6-3_Probe | GCCAAGGACCCATGAAA |
| DENV1v6-3_Rp | GCCAAAATTCCTGCTGTTGG |
| DENV2v5-1_Fp1 | AGTCAGGTCGGATYAAGCC |
| DENV2v5-1_Probe | CTATGCTACCTGTGAGCCC |
| DENV2v5-1_Rp1 | AGCTTCCATGGTTTGTGGCC |
| ***DENV2v5-2_Fp1*** | ***CGTGTCRACTGTRCAACAG*** |
| ***DENV2v5-2_ Probe*** | ***ATTCTCACTTGGAATGCTGC*** |
| ***DENV2v5-2_Rp*** | ***ARTATCCCTGCTGTTGGTG*** |
| DENV2v5-3_Fp | RCTATGCTACCTGTGAGCC |
| DENV2v5-3_ Probe | AGGCCACAAACCATGGAA |
| DENV2v5-3_Rp | CGCTAGTCCACTACGCCATG |
| DENV3v5-1_Fp | GCATGGAATGTGTGGGAGGTG |
| DENV3v5-1_ Probe | ACATATGGCTGAAACTCCGA |
| DENV3v5-1_Rp | CTGCCGACATTAGCCTATG |
| ***DENV3v5-2_Fp*** | ***AACACTCTGGGAAGGATCACC*** |
| ***DENV3v5-2_ Probe*** | ***TTGGAACACCACGATAGCT*** |
| ***DENV3v5-2_Rp*** | ***AGCAAGCCCAGCTCCTG*** |
| ***DENV***3v5-3_Fp | CGCGTGAGAAACCGTGTG |
| DENV3v5-3_Probe | TGGATCACAGTTGGCGAA |
| DENV3v5-3_Rp1 | CTCCTGCTGTTGGTGGAATG |
| DENV4v5-1_Fp1 | CTTACTTCTGGCGGCTTAC |
| DENV4v5-1_ Probe1 | GATCTGTCACTAGAGAAGGCC |
| DENV4v5-1_Rp | CCGTATGGAGAAAGAGCCATC |
| DENV4v5-2_Fp1 | CATGCTGACATGGGTTATTGG |
| DENV4v5-2Probe | CAATGGAGTGCTGGAAAGC |
| DENV4v5-2_Rp1 | CCTGGCGGTAATTGTGCTG |
| DENV4v5-3_Fp | CTGGGAGAGTGATAGACCCTAG |
| DENV4v5-3_ Probe | AGATGCCTCAAGCCAGTT |
| DENV4v5-3_R1 | CTTCCTATTCGCCCTCTTC |
| DENV4v6-2_Fp | GATCTCAGGAAGGAGCCATG |
| DENV4v6-2_Probe | AGCCACAGAAGTGGACT |
| DENV4v6-2_Rp | GCTGTGTTTCTGCCATCTC |
| ***DENV4v6-3_Fp*** | ***GCAGGCAAAAGCCACAAG*** |
| ***DENV4v6-3_Probe*** | ***AGTGGACGGGATAACAGT*** |
| ***DENV4v6-3_Rp*** | ***CATGACCTGCCCTAATTG*** |
| DENV4v6-4_Fp | GAGAGAAACCGCGTATCAACC |
| DENV4v6-4_Probe | CTTACGGATGGTGCTAGC |
| DENV4v6-4_Rp | CAGAATCCCTGCTGTTGGTG |
| ***CHIKV_FS-1_Fp1*** | ***CAGATGGCAACGAACAGG*** |
| ***CHIKV_FS-1_Probe1*** | ***CCTTTGCAAGCTCCAGATC*** |
| ***CHIKV_FS-1_Rp1*** | ***GGGTCCTCTGAGCTTCTC*** |
| CHIKV_FS-3_Fp1 | CTAGACATGGTGGACGGG |
| CHIKV_FS-3 _Probe1 | CCCATTCCAGAACACACTAC |
| CHIKV_FS-3_Rp1 | CTCATCTGTGTGACGTTGC |
| ZIKV3-1_Fp1 | ACCTCCAAGATGGCCTCATAG |
| ZIKV3-1_ Probe1 | GGGAGAGTTCAAGCTTAGGAC |
| ZIKVv3-1_Rp1 | CATGAGTTCCACAAAGGTCTTC |
| ZIKV3-2_Fp1 | GCATTTGAAGCCACTGTGAGAGG |
| ZIKV3-2_ Probe1 | GGCAAGGGCATCCATCAAA |
| ZIKV3-2_Rp1 | GTGAGAACCAGGACATTCCTCC |
| ZIKV3-3_Fp1 | CCAGAAGAGAACGGCAGCTG |
| ZIKV3-3_ Probe | TGAAGAACCCTGTTGTGGA |
| ZIKV3-3_Rp | CAATGTCAGTCACCACTATTCC |
| ***ZIKV3-4_Fp*** | ***AGGGACCTCCGACTGATG*** |
| ***ZIKV3-4_ Probe*** | ***GAAAGGGAGAATGGATGACC*** |
| ***ZIKV3-4_Rp*** | ***CCTCAATCCACACTCTGTTC*** |
